# Reverse Transcription Recombinase Polymerase Amplification Assay for the Detection of Middle East Respiratory Syndrome Coronavirus

**DOI:** 10.1371/currents.outbreaks.62df1c7c75ffc96cd59034531e2e8364

**Published:** 2013-12-12

**Authors:** Ahmed Abd El Wahed, Pranav Patel, Doris Heidenreich, Frank T. Hufert, Manfred Weidmann

**Affiliations:** Department of Virology, University Medical Centre, Goettingen, Germany; Department of Virology, Faculty of Veterinary Medicine, Mansoura University, Mansoura, Egypt; Centre for Biological Threats and Special Pathogens, Robert Koch Institute, Berlin, Germany; Department of Virology, University Medical Centre, Goettingen, Germany; Department of Virology, University Medical Centre, Goettingen, Germany; Department of Virology, University Medical Centre, Goettingen, Germany

**Keywords:** MERS-coronavirus, point-of-care assay, RPA

## Abstract

The emergence of Middle East Respiratory Syndrome Coronavirus (MERS-CoV) in the eastern Mediterranean and imported cases to Europe has alerted public health authorities. Currently, detection of MERS-CoV in patient samples is done by real-time RT-PCR. Samples collected from suspected cases are sent to highly-equipped centralized laboratories for screening. A rapid point-of-care test is needed to allow more widespread mobile detection of the virus directly from patient material. In this study, we describe the development of a reverse transcription isothermal Recombinase Polymerase Amplification (RT-RPA) assay for the identification of MERS-CoV. A partial nucleocapsid gene RNA molecular standard of MERS-coronavirus was used to determine the assay sensitivity. The isothermal (42°C) MERS-CoV RT-RPA was as sensitive as real-time RT-PCR (10 RNA molecules), rapid (3-7 minutes) and mobile (using tubescanner weighing 1kg). The MERS-CoV RT-RPA showed cross-detection neither of any of the RNAs of several coronaviruses and respiratory viruses affecting humans nor of the human genome. The developed isothermal real-time RT-RPA is ideal for rapid mobile molecular MERS-CoV monitoring in acute patients and may also facilitate the search for the animal reservoir of MERS-CoV.

## Introduction

The analysis of samples in Europe for a case of severe pulmonary syndrome and renal failure in Saudi Arabia led to the isolation and characterization of a novel human betacoronavirus termed MERS-CoV 1 . As of November 26^th ^68 deaths of 160 laboratory confirmed cases have been recorded by the WHO mainly from countries in the Middle East 2. The detection of MERS-CoV infected cases depends primary on sensitive real-time RT-PCR 3 . In developing countries, facilities for performing real-time RT-PCR are limited to centralized laboratories. The time to result can therefore be long and in many cases last up to several days. A portable, rapid, cheap, and sensitive test is needed to identify infected acute cases and would help to improve the onsite search for animal reservoirs.

In recent years a variety of isothermal amplification methods have been developed which offer the possibility of developing rapid nucleic acid detection assays for simple point-of-care systems. The recombinase polymerase amplification (RPA) assay is an isothermal amplification technology 4. RPA assays run at 42°C for 15 minutes 5 ^,^ 6 ^,^ 7 ^,^ 8. The amplification in the RPA reaction depends on binding of the recombinase to oligonucleotide primers. The complex then scans the template DNA for the corresponding sequence and initiates 5´-strand invasion of the oligonucleotide at the site of homology. The strand invasion is stabilized by the single strand binding protein. The extension of the primer ensues via a strand displacing DNA polymerase 6 ^,^ 7 ^,^ 8. Real-time detection is possible *via* an exo-probe system 5 ^,^ 6 ^,^ 7 ^,^ 8. In this study, MERS-CoV RT-RPA assay was developed to amplify and detect in real-time a fragment of the nucleocapsid gene (NC) of MERS-CoV.

## Materials and Methods

**Viral RNA**. Viral RNA are listed in table 1. MERS-CoV was provided by Ron Fouchier, Erasmus University, Rotterdam, Netherlands and the sample preparation was performed at the Robert Koch-Institut in Berlin, Germany under the ECDC Framework Service Contract Ref. No. ECDC/2008/011. The respiratory viral RNA panel for cross reactivity was provided by Landesgesundheitsamt Niedersachsen, Germany and Brunhilde Schweiger, National Reference Center for influenza viruses, Robert Koch Institute, Berlin, Germany.

**Table 1. Viral RNA tested.** The MERS-CoV RT-RPA assay only detected the MERS-CoV RNA, but not other viruses causing similar clinical picture. C_T_, cycle threshold; TT, threshold time in minute.

**Sample** **** ****	**RNA real-time RT-PCR specific assay**		**** **RT-RPA**
	**Reference** ****	**Specific assays (** **Mean Ct)**	
			**TT [min]**
**Coronaviruses**			
MERS-CoV	13	36.10	6.70
229E	14	12.6	Negative
NL63	15	14.06	Negative
OC43	14	22.99	Negative
SARS	16	14.21	Negative
**Respiratory Viruses**			
A/California/04/2009 H1N1	18	23.69	Negative
A/Wellington/1/04 H3N2		25.5	Negative
A/dk/Germany R603/06 H5N1		24.4	Negative
B/Malasiya/2506/04 (Victoria lineage)		21.2	Negative
B/Iangsu/10/03 (Yamagata lineage)		24.5	Negative
Parainfluenza virus 1 (patient isolate)	17	28.09	Negative
Parainfluenza virus 2 (patient isolate)		27.81	Negative
Parainfluenza virus 3 (patient isolate)		29.13	Negative
Parainfluenza virus 4a (patient isolate)	In-house test	21.39	Negative
Parainfluenza virus 4b (patient isolate)		28.05	Negative
Respiratory syncytial virus A	Provided by National Reference Center for influenza viruses, Robert Koch Institute, Germany		Negative
Respiratory syncytial virus B			Negative
Human rhinovirus A 89			Negative
Human rhinovirus A 1B			Negative
Human rhinovirus B 37			Negative

**Real-time RT-PCR and RT-RPA amplicon design. **Using the MERS-CoV sequence (GenBank accession number: JX869059), we designed NC real-time RT-PCR primers and Taqman probe, and RT-RPA primers and an exo-probe (Table 2) to amplify the NC gene. All oligonucleotides were produced by TIBMOLBIOL, Berlin, Germany.

**Table 2. Primers and probes for the construction of molecular standard, NC real-time RT-PCR and RT-RPA assay:** COR12 UP/DP are NC gene amplification primers; COR12 NC FP/RP/P, NC real-time RT-PCR primers and Taqman probe (FAM/TAMRA); COR12 RT-RPA FP/RP, RPA forward and reverse primer; COR12 RT-RPA P, exo-probe; BTF, B: thymidine nucleotide carrying Blackhole quencher-1, T: tetrahydrofuran spacer, F: thymidine nucleotide carrying Fluorescein; Phosphate, 3’phosphate to block elongation.

**Name** ****	**Primers and probes ** ****
COR12 UP	AATGATTCAGCTATTGTTACACAATTCG
COR12 DP	ATCTTTCTTAGTGATTACTTTTGGCTGC
COR12 NC FP	CAATAGTCAATCATCTTCAAGAGCCTC
COR12 NC RP	GGAGAAGTGCCGCGGGTA
COR12 NC P	AAACTCTTCCAGATCTAGTTCACAAGGTTCAAGATC
COR12 RT-RPA FP	AACTTCCACATTGAGGGGACTGGAGGCAA
COR12 RT-RPA RP	AGAGTTTCCTGATCTTGAACCTTGTGAACT
COR12 RT-RPA P	TCTTCAAGAGCCTCTAGCTTAAGCAGAAAC-**BTF-**TCCAGATCTAGTTC-**P**

**RNA standard and RT-RPA conditions.** The NC gene was amplified using COR12 UP and COR12 DP (Table 2) (nt 28989-29291 of GenBank accession number: JX869059). The amplificate was ligated into plasmid pCRII (Invitrogen, Darmstadt, Germany). RNA was transcribed, quantified, diluted into a dilution range from 10^7^ to 10^1^ RNA molecules/µl and tested by real-time RT-PCR as described 10. RT-RPA was performed in a 50 µl volume using the TwistAmp™ exo kit (TwistDx, Cambridge, UK) 420 nM RPA primers, 120 nM exo-probe, 2 µM DTT, 14 mM magnesium acetate, TwistAmp^TM^ rehydration buffer, 2 U Transcriptor (Roche, Mannheim, Germany), 20 U RiboLock RNase inhibitor (Fisher, Schwerte, Germany), and 19 mM DTT (Roche). All reagents except for the template or sample RNA and magnesium acetate were prepared in a mastermix, which was distributed into each tube of the 0,2 ml reaction eight-tube strip containing a dried enzyme pellet. Magnesium acetate was pipetted into the tube lids. Subsequently, 5 µl sample was added to the tubes. The lids were closed and the magnesium acetate centrifuged into the tubes using a minispin centrifuge and the tubes immediately placed into the tubescanner device (Qiagen Lake Constance, Stockach, Germany). Fluorescence measurements were performed in an ESEquant tubescanner at 42°C for 10 minutes. A combined threshold and signal slope analysis were used for signal interpretation which can be confirmed by 2^nd^ derivative analysis (Tubescanner studio software, Qiagen Lake Constance, Stockach, Germany).

**Determination of sensitivity and specificity.**The sensitivity of the NC real-time RT-PCR assay and the RT-RPA assay was tested using the quantitative RNA standard in 8 replicates, the threshold time was plotted against molecules detected and a semi-log regression was calculated using the Prism software (Graphpad Software Inc., San Diego, California) and a probit regression were calculated using the Statistica software (StatSoft, Hamburg, Germany).

Sensitivity was additionally evaluated using RNA extracts from dilutions of MERS-CoV virus culture supernatant. The supernatant containing 6.3 x 10^8^ genome equivalents/ml (ge/ml) as determined by the UpE real-time RT-PCR 13 was diluted in tenfold steps. RNA was extracted from these dilutions using the (QIAamp viral RNA mini kit, Qiagen, Hilden, Germany) and eluted in 200 µl nuclease free water. A volume of 2.5 µl were tested simultaneously in three real-time RT-PCR assays (UpE 13 , Orf1A 3, NC) and the RT-RPA assay. For cross detection studies RNA of coronaviruses and other respiratory viruses listed in table 1 were tested.

## Results

A 303 nt standard RNA was produced via in *vitro* transcription from a plasmid containing a NC gene fragment of MERS-CoV. The sensitivity was determined using a dilution range of 10^7^-10^1^ RNA molecules/µl of the MERS-CoV RNA standard. The NC real-time RT-PCR and the RT-RPA assay showed a sensitivity of 10^1^ RNA molecules detected (Figure 1 and 2). The probit regression predicted a sensitivity of 21 and 10 RNA molecules respectively for RT-RPA (figure 3) and NC RT-PCR assay.

**MERS-CoV RT-RPA assay.**
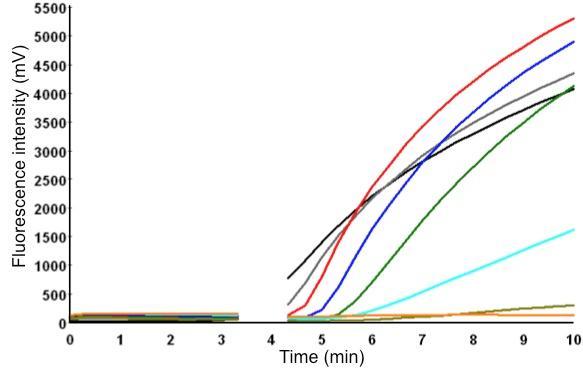
Over time development of fluorescence using a dilution range of 10^7^-10^1^ molecules/µl of the RNA molecular standard (Tubescanner Studio Software, Qiagen, Germany). MERS-CoV RT-RPA assay sensitivity is 10 copies and yielded results in maximum 7 minutes. 10^7^ represented by black line; 10^6^, gray; 10^5^, red; 10^4^, blue; 10^3^, green; 10^2^, cyan; 10^1^, dark khaki; negative control, orange. No fluorescence signals was measured for one minute (after 3 minutes of the start of the reaction) because of the need of mixing the content.

**Analytical sensitivity of MERS-CoV NC real-time RT-PCR and RT-RPA.**
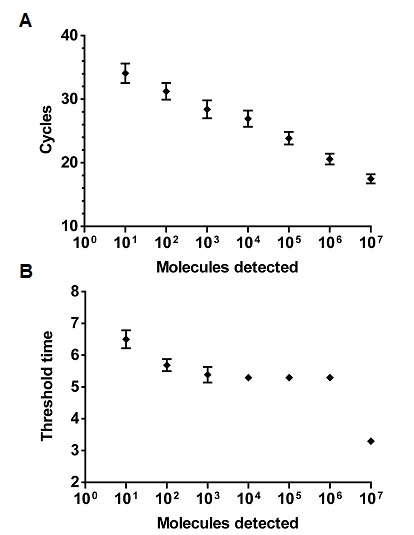
The analytical sensitivity was determined on RNA molecular standard (8 runs) for real-time RT-PCR (A) and real-time RT-RPA (B). Both assays have a sensitivity of 10 RNA molecules. The RT-RPA assay was much faster than the RT-PCR as the run time of the RT-RPA is between 3-7 minutes for 10^7 ^and 10^1^molecules, respectively. While the RT-PCR needed up to 2 hours. Consequently, RT-PCR results is linear, while RT-RPA not.

**Probit regression analysis of MERS-CoV RT-RPA assay:**
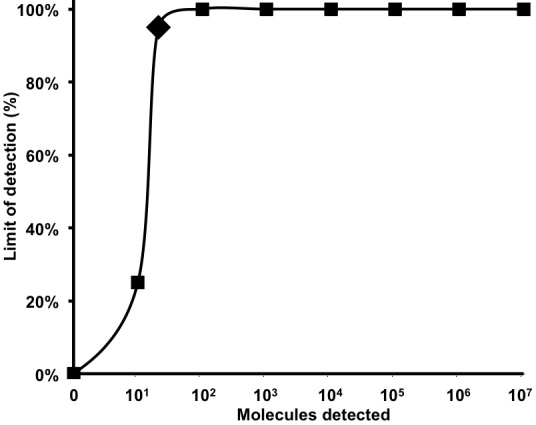
The probit analysis was performed using Statistica software on data of the eight runs of 10^7^-10^1 ^RNA molecular standard. The limit of detection at 95% probability (21 RNA molecules) is depicted by a Rhomboid.

Using a ten-fold serial dilution of RNA extracted from MERS-CoV tissue culture supernatant, the UpE real-time RT-PCR assays 13 detected down to a dilution of 10^-6^, whereas the Orf1A 3 and NC real-time RT-PCR assays as well as the RT-RPA assay detected RNA down to a dilution of 10^-5^, corresponding to 7.5 genome equivalents/reaction (3000 genome equivalents/ml) (Table 3). The RT-RPA assay specificity was determined using several coronaviruses and respiratory viruses (Table 1) as well as human genome. None of them were detected.

**Table 3. Sensitivity of RT-RPA assay in comparison with three real-time RT-PCR assays:** Ten-fold serial dilution of RNA extracted from MERS-CoV culture supernatant was used. UD is undetected; C_T_, cycle threshold; TT, threshold time in minute

**Genome equivalence/reaction **	**Real-time RT-PCR**			**RT-RPA**
	**UpE ** 13	**Orf1A ** 3	**NC**	
	**Cт Mean**	**Cт Mean**	**Cт Mean**	**TT Mean**
9.92E+04	21.97	23.93	22.02	4.30
1.23E+04	25.10	27.14	24.84	4.70
1.30E+03	28.58	31.32	28.37	5.00
9.66E+01	32.50	36.49	31.63	5.50
7.5E+00	36.43	40.91	36.29	6.30
1.14E+00	39.76	UD	UD	UD

## Discussion

The MERS-CoV causing a severe respiratory disease erupted in the Near East last year. In outbreak situations, rapid diagnostic tools can generally help to confirm infection in suspected patients and to test contacts in order to contain spread of the infectious agent. For wide scale testing, robust field-deployable test systems provide an advantage and allow testing in make shift laboratories or at an outbreak site.

The performance of the isothermal RT-RPA assay for the detection of MERS-CoV RNA described here equals that of a published real-time RT-PCR assay 3 in terms of sensitivity and specificity at a run time of only 10 minutes. RNA concentrations in samples collected from MERS-CoV-infected patient between days 11-16 of infection were 1.2 x10^6^, 5370, 2691, and 1031, for lower respiratory tract, oronasal swabs, urine, and stool, respectively 11. Therefore, the detection limit of our RT-RPA assay (10 RNA copies) is ideal for identifying MERS-CoV. Unfortunately, no clinical samples were available in our hand to test.

The run time of the RT-RPA was 10 minutes (figure 1 and 2B), and including nucleic acid extraction from samples, results can therefore be obtained in about 30 minutes. Reagents are available as dried pellets (cost 4 Euro/test) and the detection device is comparably cheap (around 4500 Euro). Rapid point-of-care diagnosis and field investigations are feasible as recent use of this technology during the FMDV outbreak in Egypt in 2012 has shown 5. We recently successfully operated a mobile RT-RPA unit consisting of a nucleic acid extraction kit, a RT-RPA kit, a pipette set, a magnetic separator, a tubescanner, and a laptop in Kedougou, Senegal using a solar panel (manuscript in preparation). The experiences made indicate that mobile RT-RPA is a very good alternative for mobile point-of-care detection, since infrastructure and equipment needs are below that of laboratory based or mobile RT-PCR 6 ^,^ 7 ^,^ 8.

During the Hajj, around three million pilgrims from allover the world visit Mecca, Saudi Arabia for an average of ten days . Physical stress on the pilgrims may increase the susceptibility to diseases 12 and the density of the susceptibles might therefore be conducive to transmission of MERS-CoV. Therefore, implementation of mobile RT-RPA units during the Hajj might be an easy tool to detect MERS-CoV infected cases on site to identify acute cases and prevent the spread of the virus. RT-RPA might also help to improve affordable monitoring for MERS-CoV by public health laboratories worldwide.
